# Virusplot: a web server for viral integration analysis and visualization

**DOI:** 10.3389/fonc.2025.1539782

**Published:** 2025-02-19

**Authors:** Erqiang Hu, Jianhong An, Adam J Gersten, Nicole Wu, Nicole Kawachi, Jing Zhu, Gregory Rosenblatt, Stelby Augustine, Richard V. Smith, Jeffrey E Segall, Harry Ostrer, Antonio L Amelio, Christine H. Chung, Michael B. Prystowsky, Thomas J. Ow, Wenjun Deng, Shanye Yin

**Affiliations:** ^1^ Department of Pathology, Albert Einstein College of Medicine, Bronx, NY, United States; ^2^ Einstein Pathology Single-cell & Bioinformatics Laboratory, Bronx, NY, United States; ^3^ Montefiore Einstein Comprehensive Cancer Center, Albert Einstein College of Medicine, Bronx, NY, United States; ^4^ Department of Otorhinolaryngology-Head and Neck Surgery, Montefiore Medical Center/Albert Einstein College of Medicine, Bronx, NY, United States; ^5^ The University of Texas at Austin, Austin, TX, United States; ^6^ Department of Tumor Microenvironment and Metastasis, H. Lee Moffitt Cancer Center & Research Institute, Tampa, FL, United States; ^7^ Department of Head and Neck-Endocrine Oncology, H. Lee Moffitt Cancer Center & Research Institute, Tampa, FL, United States; ^8^ Department of Neurology, Massachusetts General Hospital, Harvard Medical School, Boston, MA, United States

**Keywords:** viral integration, genomic visualization, hotspot analysis, computational biology, bioinformatics tool, host-virus interaction

## Abstract

The integration of viral DNA into the human genome is a critical event in the pathogenesis of various cancers. This process leads to genomic instability, disrupts cellular regulatory mechanisms, and activates oncogenes or inactivates tumor suppressor genes. Despite significant advancements in genome sequencing technologies, there remains a notable lack of computational tools, particularly web-based applications, specifically designed for viral integration analysis and visualization. To address this gap, we present virusPlot, a web server with the following functional modules: (i) automatic retrieval of virus genome sequences and their annotation; (ii) visualization of virus integration locations and read counts through a graphical representation that links viral and host genome integration sites, facilitating the interpretation of integration patterns; (iii) analysis of virus integration hotspots using Fisher’s exact test; and (iv) integration of various functions into an interactive web platform via shinyapp. VirusPlot efficiently processes and visualizes integration data from viruses and host genomes, providing researchers with an intuitive and user-friendly analytical tool that simplifies the complexity of virus integration analysis.

## 1 Introduction

Viral insertion into the host genome is a critical event in the viral life cycle of several viruses associated with tumorigenesis (1, 2). Specifically, viruses, such as human papillomavirus (HPV) (3, 4), hepatitis B virus (HBV) (5), Epstein-Barr virus (EBV) (6), and Human T-cell leukemia virus type 1 (HTLV-1) (7), are well-known for their oncogenic potential through this mechanism. The integration of viral DNA can result in genomic alterations that drive malignant transformation, contributing to cancer development and progression (1, 8–12). Understanding the molecular mechanisms of viral integration and its impact on cellular pathways is essential for developing targeted therapies and preventive strategies against virus-associated cancers.

Recent advances in genome sequencing methods have significantly enhanced the detection of viral integration events in tumor genomes. Whole Genome Sequencing (WGS) provides a comprehensive view of the entire tumor genome and allows for the identification of viral integration sites across the genome (3, 13–15). Capture sequencing involves the enrichment of viral sequences and their adjacent host sequences before sequencing, which is highly effective for detecting viral integration sites and mapping the integration landscape with high sensitivity and specificity (14, 16, 17). The development of long-read sequencing technologies offers the advantage of reading longer DNA fragments, which can span entire integration sites and provide more accurate mapping of integration events (18–21). This method is particularly useful for resolving complex integration events and structural variations.

Additionally, tools such as isling (22) and Vseq-Toolkit (23) have been developed for viral integration analysis, along with other tools benchmarked in these studies, providing a foundation for comparative assessments.

Viral integration events are complex and require sophisticated algorithms to accurately detect and interpret insertion sites within the host genome (9). These events can vary significantly in their genomic context, integration frequency, and impact on gene regulation, necessitating specialized tools for comprehensive analysis (1, 3, 9, 14). Currently, most available tools for viral integration analysis are either standalone software or scripts requiring a high level of bioinformatics expertise to operate (24–26). These tools often demand substantial computational resources and can be challenging for researchers without advanced programming skills. Furthermore, effective visualization tools are lacking, which are essential for interpreting the results of viral integration studies, allowing researchers to explore integration sites, their genomic contexts, and potential effects on gene expression and genome stability.

The absence of user-friendly, web-based platforms limits accessibility and hinders the widespread adoption of viral integration studies in the broader research community. To address this, we have developed virusPlot, an all-in-one analysis and visualization software. Key features of virusPlot include the automatic retrieval of virus genome sequences and annotation information, a visualization tool that represents viral integration events by connecting integration sites in the host genome with corresponding positions in the viral genome, and virus integration hotspot analysis using Fisher’s exact test to help identify possible integration hotspot regions. Importantly, virusPlot integrates all these functions into an interactive web platform via shinyapp, expanding accessibility to users without technical or computational skills. The integration of interactive, web-based visualization tools can significantly enhance the ability to communicate findings, generate hypotheses, and facilitate collaborative research.

## 2 Methods and functions

**Figure 1:**
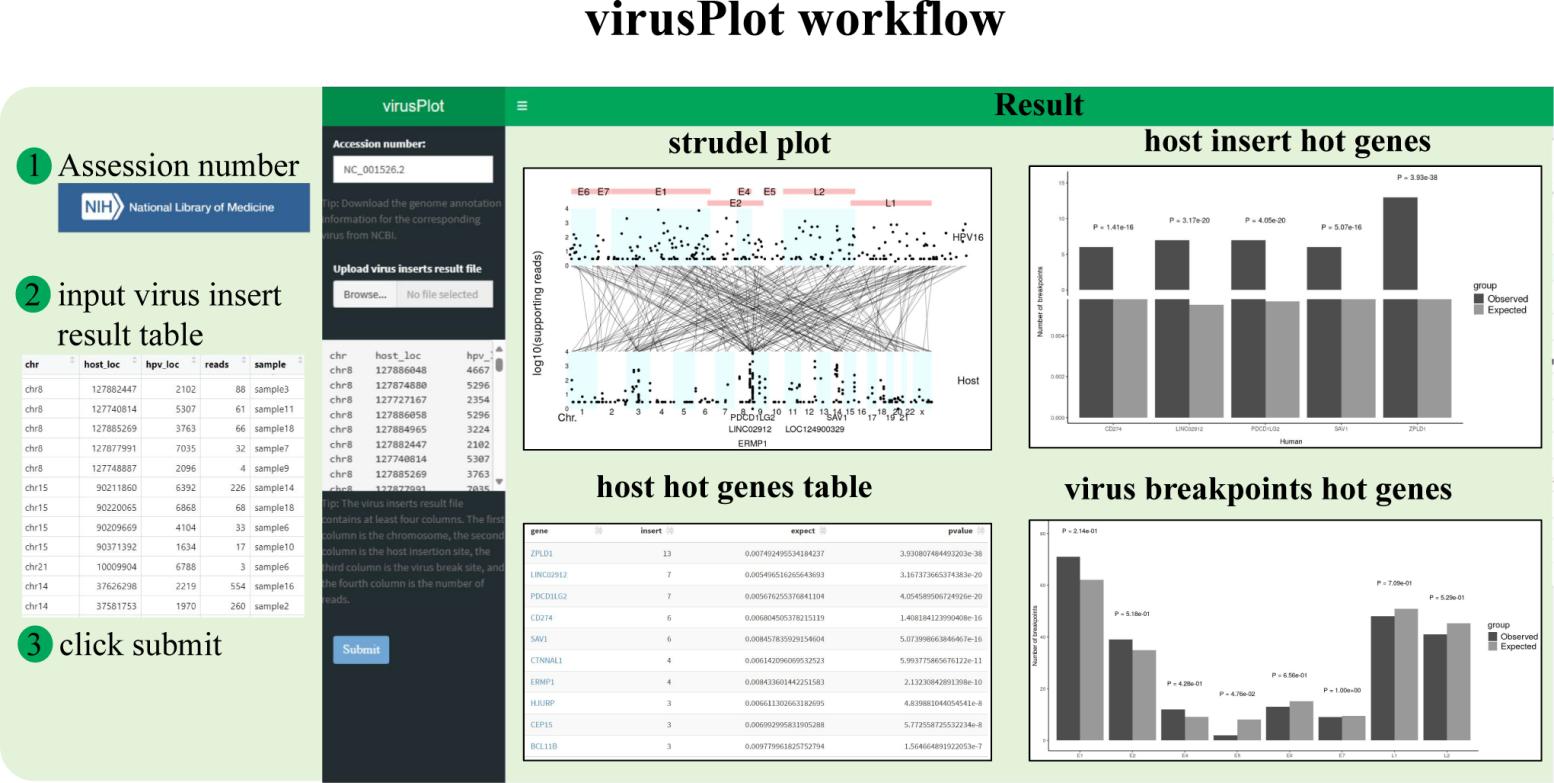
Screenshot of the virusPlot web tool. This package encompasses four main functionalities: (i) Automatic retrieval of virus genome information, (ii) Virus integration information visualization, (iii) Virus integration hot spots analysis, hot spots refer to genes with a statistically significant number of viral integration events, identified using Fisher’s exact test or Chi-square test, and (iv) a user-friendly web application interface (shinyapp).

**Figure 2:**
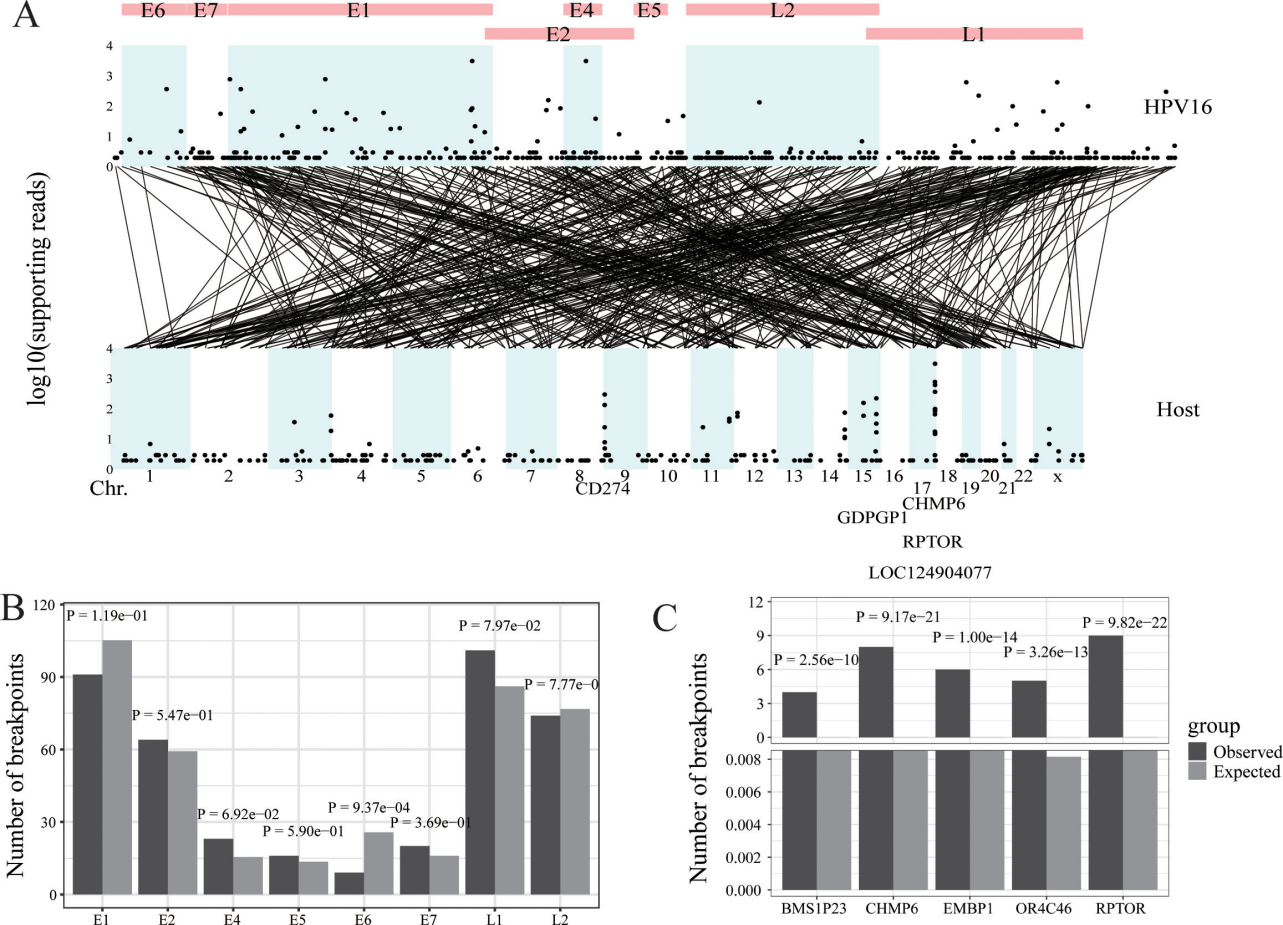
HPV integration in oropharyngeal cancer. **(A)** Strudel plot of HPV16 integration information. **(B)** The HPV16 integration hotspot analysis of the HPV16 genome. **(C)** The HPV16 integration hotspot analysis of the host genome.

**Figure 3:**
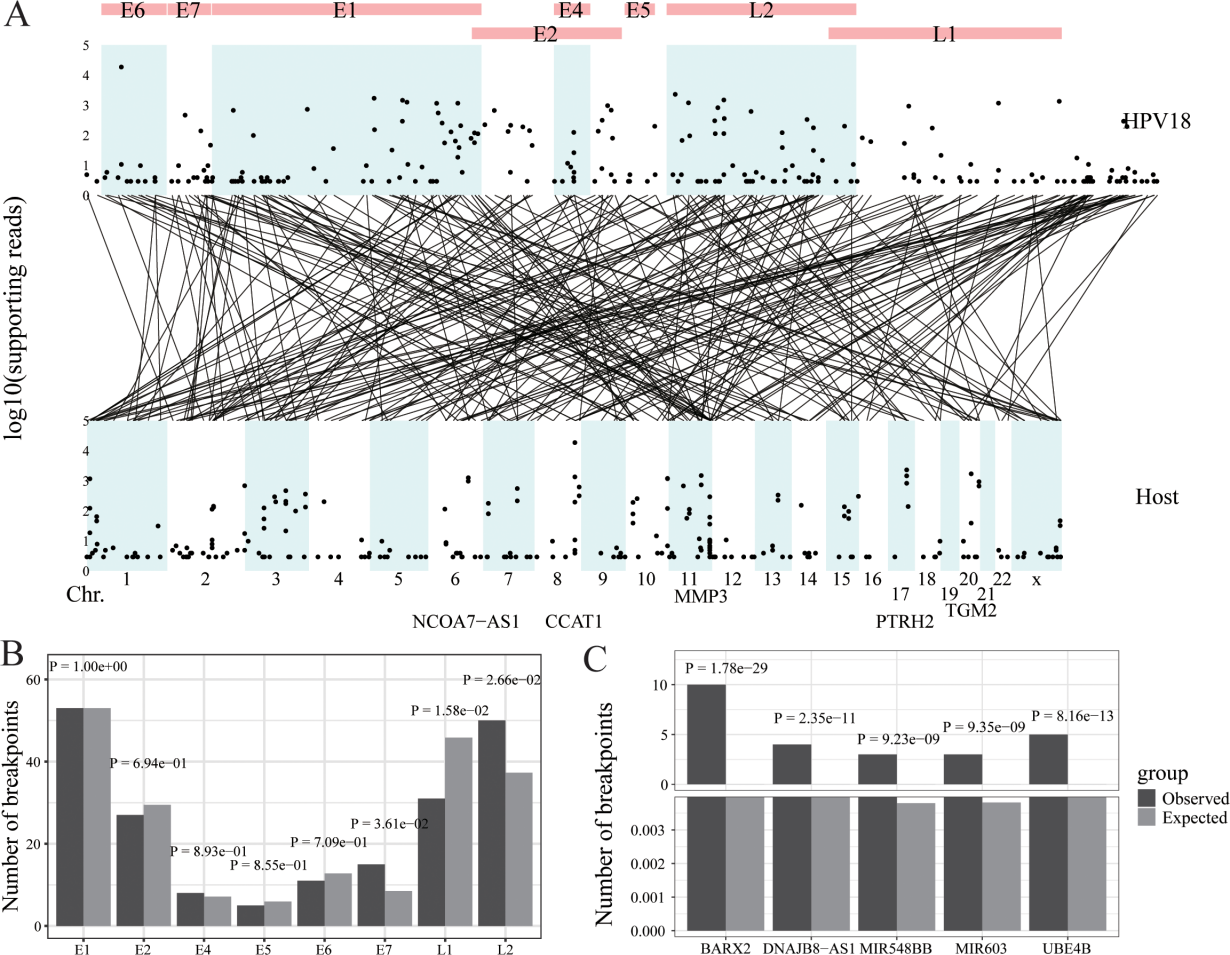
HPV integration in cervical cancer. **(A)** Strudel plot of HPV18 integration information. **(B)** The HPV18 integration hotspot analysis of the HPV18 genome. **(C)** The HPV18 integration hotspot analysis of the host genome.

**Figure 4:**
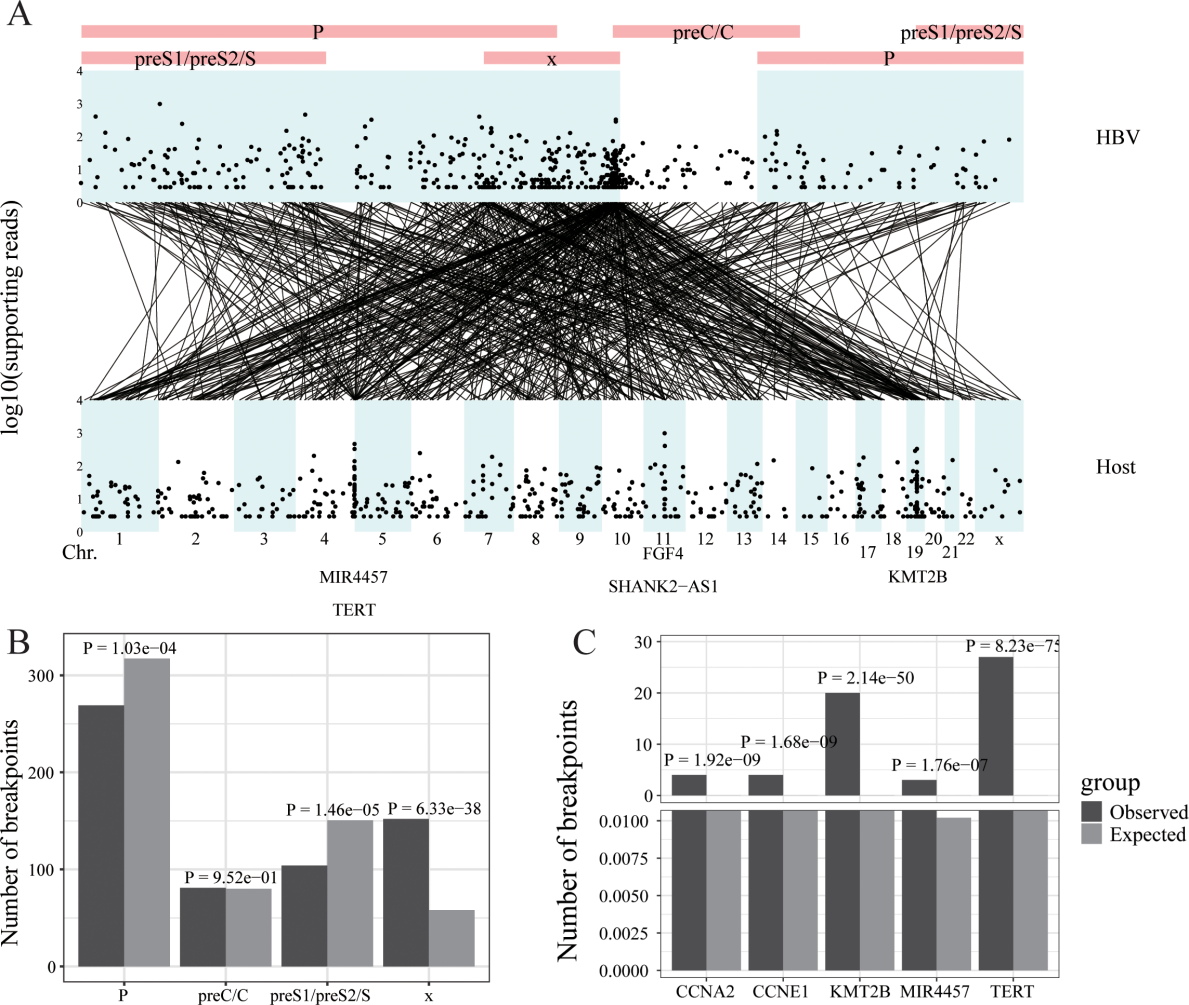
HBV integration in liver cancer. **(A)** Strudel plot of HBV integration information. **(B)** The HBV integration hotspot analysis of the HBV genome. **(C)** The HBV integration hotspot analysis of the host genome.

We have developed virusPlot, an R package that offers a comprehensive suite of tools for analyzing and visualizing virus integration into the host genome. The user interface and back-end of virusPlot are built using Shiny. Analysis results are displayed on the web page and can be downloaded in various formats, including PDF, PNG, EPS, TXT, and HTML (for more details, refer to the website help pages). The workflow and typical output schema are illustrated in **Figures 1**–**4**. Detailed functions and operations for each module are described below.

### 2.1 Automatic retrieval of virus genome

The virusPlot package includes two key functions for retrieving virus genome data: get_virus_genom and get_virus_annotation. The get_virus_genom function automates the process of obtaining viral genomic sequences from the National Center for Biotechnology Information (NCBI) database. By leveraging NCBI’s extensive repository, this function provides users with accurate and up-to-date viral sequences for their research. Complementing this, the get_virus_annotation function retrieves gene annotation information for the obtained viral genomes, which is essential for understanding the functional roles of various viral genes and their potential impact on the host organism.

### 2.2 Quality control

To ensure the robustness of the input data analysis, we have incorporated a rigorous quality control mechanism. This mechanism allows users to apply filters based on two adjustable parameters: minimum read number and p-value threshold. For example, users can set a minimum read number (e.g., ≥10 reads) to exclude low-confidence breakpoints supported by insufficient reads, and a p-value threshold (e.g., p < 0.05) to ensure statistical significance. These parameters can be fine-tuned based on the specific characteristics of the dataset, such as sequencing depth or experimental design.

We have observed that adjusting these parameters has distinct effects on the dataset:

- Increasing the minimum read number threshold improves the confidence of the retained breakpoints but may reduce the overall number of breakpoints available for downstream analysis.
- Tightening the p-value threshold enhances statistical rigor but might exclude potentially relevant breakpoints with weaker statistical signals.

### 2.3 Virus integration information visualization

A novel visualization approach, the strudel plot, has been developed within the virusPlot package to graphically represent the complex integration patterns of viruses within host genomes. The strudel plot is a multi-faceted structure that effectively displays integration sites, viral genome breakpoints, and the correspondence between host integration sites and viral breakpoints. The plot is organized into five distinct components:

- Viral Genome Retrieval: Presents the sequence and annotated information of the viral genome.
- Viral Integration Sites: Illustrates the positions of viral integration sites along with the number of associated reads.
- Integration Breakpoints: Maps the breakpoints in both the viral and host genomes.
- Host Integration Sites: Shows the positions of integration sites in the host genome along with their respective read counts.
- Host Gene Hotspots: Identifies the host genes that are hotspots for viral integration.

VirusPlot analyzes viral integration breakpoints irrespective of the completeness of the viral genome at the site. The analysis supports both forward and reverse orientations of the virus and incorporates rearranged fragments by identifying and visualizing integration breakpoints on both the viral and host genomes.

### 2.4 Virus integration hot spots analysis

Viral integration hotspots are pivotal for comprehending virus-induced cancer mechanisms and for devising targeted therapies and preventive strategies. Identifying these hotspots holds promise for early detection, prognostic insights, and personalized treatment approaches. In the virusPlot package, a robust analysis tool is included for detecting viral integration hotspots within the host genome. A gene is identified as a potential hotspot if the observed number of integration sites significantly exceeds the expected count by chance. This comparison, facilitated by both Fisher’s exact and chi-square tests, offers valuable insights into the preferential integration patterns of viruses, critical for deciphering viral persistence mechanisms and pathogenesis. The expected number of integration sites for each genomic region was calculated as follows:


$$\text{Excepted count }=\text{ Total observed integrations }\times \frac{\text{Region size }\left(\text{bp}\right)}{\text{Total genome size }\left(\text{bp}\right)}$$

Statistical significance was determined using Fisher’s exact test and Chi-square test by comparing the observed and expected counts. The p-value threshold for significance was set at 0.05.

The web-based version of VirusPlot processes viral integration data using the hg38 human genome assembly by default. For users of the R package version, the genome assembly can be customized by specifying the TxDb parameter in functions such as get_hot_gene and strudel_plot. This flexibility allows users to work with data aligned to different genome versions, ensuring compatibility with their specific datasets and annotations.

### 2.5 A user-friendly web application interface

To make virusPlot widely accessible and easy to use, we’ve integrated a shinyapp, providing a user-friendly web interface for accessing all features without the need for coding. This web app simplifies operations, requiring no local setup or specific hardware, as all computations are cloud-based, ensuring compatibility across different systems. Through practical case studies using our own data and published HPV and HBV integration data in various cancers (i.e., oropharyngeal cancer, cervical cancer and hepatocellular cancer) (**Figures 2**–**4**), virusPlot has proven effective in displaying integration sites and identifying hotspots, underscoring virusPlot’s utility in simplifying the complex analysis of viral integration and providing valuable insights into the genomic alternations and regulatory disruptions associated with virus-related cancers.

### 2.6 A data security and privacy

VirusPlot employs multiple measures to ensure the security of user data. All data transfers between the user and the server are encrypted using HTTPS. Uploaded data is processed in-memory without being stored permanently on the server, and all data is automatically deleted upon completion of the analysis. The server is hosted on a secure platform with firewalls and regular updates to protect against vulnerabilities.

## 3 Case study

### 3.1 HPV integration in oropharyngeal cancer (Data in this study)

Whole Exome & HPV capture sequencing (WEHS) was performed on 20 head and neck cancer samples. We used the Survirus software (24) to detect HPV16 integration information. This analysis revealed 471 integration sites and 13,007 reads encompassing these sites. The viral integration sites were evenly distributed throughout the HPV16 genome but concentrated in specific chromosomal regions of the host genome, such as chr17, chr9, and chr15. The five genes with the highest number of viral read insertions were CHMP6, RPTOR, LOC124904077, CD274, and GDPGP1 (**Figure 2A**). Regarding the HPV16 genome, fewer integration sites were observed in the E6 gene compared to random occurrences, while no significant differences were noted for other genes, and no hotspot integration genes were identified (**Figure 2B**). In the host (human) genome, several hotspots for HPV16 insertion were identified, including BMS1P23, CHMP6, EBMP1, OR4C46, and RPTOR, compared to random occurrences (**Figure 2C**).

### 3.2 HPV integration in cervical cancer

Ma’s Lab conducted DNA sequencing on 39 cervical cancer samples collected from Tongji Hospital in Wuhan and Jingmen No. 2 People’s Hospital in Hubei Province, China, between 2007 and 2014 (14). Exfoliated cervical epithelial cells were collected using cervical brushes. The high-throughput Viral Integration Detection (HIVID) (27) was then applied to these samples to detect HPV18 integration information. This analysis identified 241 integration sites and 48,603 reads including these sites. The viral integration sites were evenly distributed across the HPV18 genome and concentrated in specific chromosomal regions of the host genome, such as chr8, chr11, and chr17. The five genes with the highest number of viral read insertions were CCAT1, PTRH2, TGM2, MMP3, and NCOA7-AS1 (**Figure 3A**). In the HPV18 genome, the actual number of integration sites in the E7 and L2 genes was significantly higher than random, indicating hotspots for integration, while the L1 gene had significantly fewer integration sites than random (**Figure 3B**). In the host (human) genome, multiple hotspots for HPV18 insertion were identified, including BARX2, DNAJB8-AS1, MIR548BB, MIR603, and UBE4B, compared to random occurrences (**Figure 3C**).

### 3.3 HBV integration in liver cancer

Wang’s Lab performed DNA sequencing on 138 hepatocellular carcinomas (HCCs) collected at the Eastern Hepatobiliary Surgery Hospital in Shanghai from 2009 to 2010 using high-throughput viral integration detection (HIVID) method (28). This analysis identified 546 integration sites and 13,242 reads encompassing these sites. The reads containing viral integration sites were primarily concentrated in the X gene of the HBV genome and on chromosomes chr5, chr11, and chr19 of the host genome. The five genes with the highest number of viral read insertions were MIR4457, TERT, FGF4, SHANK2-AS1, and KM12B (**Figure 4A**). Regarding the HBV genome, the actual number of integration sites in the X gene was significantly higher than random, indicating hotspots for integration, whereas the PreS1/PreS2/S and P genes had significantly fewer integration sites than random (**Figure 4B**). In the host (human) genome, multiple hotspots for HBV insertion were identified, including TEAR, KMT2B, CCNE1, CCNA2, and MIR4457, compared to random occurrences (**Figure 4C**).

## 4 Discussion

The virusPlot platform offers a comprehensive suite of tools and workflows designed to simplify the analysis and visualization of viral integration events within host genomes. Serving as an visualization solution, virusPlot streamlines the process of identifying integration hotspots and understanding the impact of viral integration on cancer development. With its intuitive interface, virusPlot enables researchers, including experimental biologists without computational programming skills, to explore complex viral integration patterns and gain valuable insights into virus-associated cancers. By integrating multiple analytical approaches and statistical tests, virusPlot enhances the analysis of viral integration data, complementing traditional methods and enabling more comprehensive investigations. As sequencing methods continue to evolve and become more cost-effective, an increasing number of viral-associated cancers and viral integration events are expected to be uncovered (1, 20). This expanding dataset of viral-related cancer genomes and integration sites will provide valuable insights into the role of viruses in oncogenesis and facilitate the development of targeted therapies and preventive strategies against virus-associated cancers. We are committed to maintaining the virusPlot platform and continuously updating it with new data and methods, ensuring its relevance and utility for the research community over the coming years. Through its user-friendly interface and powerful analytical capabilities, virusPlot aims to accelerate discoveries in viral oncology and facilitate the identification of novel cancer pathways and therapeutic targets.

To evaluate the usability of VirusPlot, we compared it with other visualization tools commonly used for integration data, such as Circos (29). Users reported that VirusPlot’s interactive interface significantly reduced the complexity of generating visualizations, compared to the manual configurations required by Circos. Furthermore, VirusPlot provides integration-specific features, such as the strudel plot and hotspot analysis, which are not available in these general-purpose tools. Additionally, the web-based accessibility of VirusPlot eliminates the need for local installations, making it a more user-friendly and efficient tool for analyzing viral integration data.

Future updates to virusPlot aim to expand its capabilities by including support for additional genomic data types, such as RNA-seq and long-read sequencing data. Planned features include new visualization tools, such as circular diagrams and VCF diagrams, which are currently under development. We also aim to integrate more advanced statistical modules to accommodate complex integration scenarios, such as those involving rearranged viral genomes.

## Data Availability

Publicly available datasets were analyzed in this study. This data can be found here: https://github.com/huerqiang/virusPlot.
